# The Function of Termicin from *Odontotermes formosanus* (Shiraki) in the Defense against *Bacillus thuringiensis* (Bt) and *Beauveria bassiana* (Bb) Infection

**DOI:** 10.3390/insects15050360

**Published:** 2024-05-16

**Authors:** Xiaogang Li, Mingyu Wang, Kai Feng, Hao Sun, Fang Tang

**Affiliations:** 1 Co-Innovation Center for Sustainable Forestry in Southern China, Nanjing Forestry University, Nanjing 210037, China; 18434763391@163.com (X.L.); myw@njfu.edu.cn (M.W.); fengkai@njfu.edu.cn (K.F.); 2 College of Forestry, Nanjing Forestry University, Nanjing 210037, China; 3 Jiangsu Province Rural Water Conservancy Science and Technology Development Center, Nanjing 210029, China; jssunh@126.com

**Keywords:** *O. formosanus*, termicin, inactivated dsRNA-HT115, Bt, Bb, biological control

## Abstract

**Simple Summary:**

*Odontotermes formosanus* (Shiraki) is a termite species recognized for its capacity to cause substantial harm to trees and buildings. Termites have co-evolved with pathogenic bacteria, leading to the emergence of a robust innate defense mechanism. Termicin, an antimicrobial peptide, plays a pivotal role in safeguarding termites against external threats. This study has successfully devised a method for the rapid production of inactivated dsRNA-HT115, thereby augmenting the efficacy of *Bacillus thuringiensis* (Bt) and *Beauveria bassiana* (Bb) against termites. Additionally, it lays the groundwork for a novel approach to termite control.

**Abstract:**

*Odontotermes formosanus* (Shiraki) is a subterranean termite species known for causing severe damage to trees and structures such as dams. During the synergistic evolution of *O. formosanus* with pathogenic bacteria, the termite has developed a robust innate immunity. Termicin is a crucial antimicrobial peptide in termites, significantly contributing to the defense against external infections. Building upon the successful construction and expression of the dsRNA-HT115 engineering strains of ds*Oftermicin1* and ds*Oftermicin2* in our laboratory, this work employs the ultrasonic breaking method to establish an inactivated ds*Oftermicins*-HT115 technological system capable of producing a substantial quantity of dsRNA. This approach also addresses the limitation of transgenic strains which cannot be directly applied. Treatment of *O. formosanus* with ds*Oftermicins* produced by this method could enhance the virulence of both Bt and Bb to the termites. This study laid the theoretical groundwork for the development of novel termite immunosuppressants and for the advancement and application of termite biological control strategies.

## 1. Introduction

Termites pose a significant threat to forest resources and crops [1]. Moreover, they can nest in dams and reservoirs, potentially leading to catastrophic dam collapses [2,3]. Termite control primarily relies on chemical control techniques, which are highly effective but can be environmentally and medically harmful to varying degrees [4]. Consequently, biological control techniques have garnered significant attention for termite control. Several pathogenic microorganisms have been identified as highly detrimental to termites in laboratory studies. Research has shown *Metarhizium anisopliae* (Ma) has a good control effect on *Odontotermes obesus* (Rambur) in the laboratory [5,6]. Likewise, *Bacillus thuringiensis* (Bt) has been shown to have a significant impact on *Microtermes obesi* (Holmgren) [7]. Moreover, entomopathogenic nematodes such as *S. riobrave* have been observed to infect *Heterotermes aureus* (Snyder), leading to high mortality rates [8]. However, the utilization of pathogenic microorganisms for termite control has been rarely reported due to the diverse immune behaviors exhibited by termites [9]. Termites possess a sophisticated defense mechanism against pathogens, encompassing both individual and social immunity [10]. This ability enables them to swiftly eliminate pathogens and prevent them from causing harm [10,11]. RNA interference (RNAi) technology has the potential to control termites by suppressing the expression of immune genes, thereby overcoming the defense mechanisms of termites. Combining pathogenic microbial control with RNAi is expected to enhance the effectiveness of termite prevention and control in practical applications, which is significant for advancing termite biological control.

RNA interference (RNAi) is a gene silencing mechanism induced by small non-coding RNA molecules consisting of 20–30 nucleotides. Initially discovered in *Caenorhabditis elegans* [12], this mechanism has been found to be ubiquitous in a broad spectrum of eukaryotic organisms, such as fungi, plants, and insects. Nevertheless, previous studies have not extensively investigated the role of RNAi in termite control. This was accomplished by injecting short interfering RNAs (siRNAs) into *Reticulitermes flavipes* (Kollar) [13]. In their study, Wu and Li et al. (2018) injected workers of *Coptotermes formosanus* (Shiraki) with dsRNA targeting the β-glucosidase genes (*CfBG*-Ia and *CfBG-Ib*), revealing a noteworthy reduction in gene expression, although no lethal effect was observed [14]. Wu et al. (2019) investigated the effects of RNAi on termites by targeting five endoglucanase genes (*GfEGs*) in workers of *C. formosanus*, revealing a noteworthy rise in the termite mortality rate, accompanied by a decrease in enzyme activity and body weight [15]. These studies provide evidence that the selection of target genes may be key to the successful application of RNAi control technology.

Targeting insect immunity genes is crucial for RNAi, given that insect immunity is primarily derived from innate immunity [16]. Insects possess innate immunity, encompassing both humoral and cellular immunity [17]. Antimicrobial peptides (AMPs) play essential roles as immune factors in the humoral immunity of insects. These peptides serve as crucial molecular barriers that protect the host against the invasion of pathogenic microorganisms. AMPs, consisting of a signal peptide and a mature peptide, are synthesized in tissues such as the fat body and hemocytes. Following synthesis, the mature peptide is released into the hemolymph through in vivo enzymatic cleavage to exert its antimicrobial effect [18]. Aspergillin, isolated from *Bombyx mori*, possesses prominent antimicrobial activity against both Gram-positive and Gram-negative bacteria [19,20]. In contrast, thanatin, an antimicrobial peptide induced and isolated from *Podisus maculiventris*, exhibits potent antimicrobial activity against both Gram-positive and Gram-negative bacteria, as well as filamentous fungi [21]. Lamberty et al. (2001) isolated two AMPs, termicin and spinigerin, for the first time in *Pseudacanthotermes spiniger*, a species of termite [22]. Furthermore, termicin has been shown to act on the cell membranes of fungi and some Gram-positive organisms, with the highest activity observed against filamentous fungi [23]. Spinigerin demonstrates effectiveness against both gram-positive and gram-negative bacteria, as well as fungi, and it is not induced by microbial infection but persists in the termite blood cavity [24,25]. AMPs play a crucial role in termite immunity against pathogen invasion. Thus, the genes associated with AMPs represent significant targets for termite RNA interference (RNAi).

*O. formosanus* is a soil-dwelling termite known for causing significant damage to trees, crops, buildings, and is particularly harmful to river dams [26]. In our laboratory, we isolated a strain of *Serratia marcescens* Bizio (SM1) from *O. formosanus*, which demonstrated high virulence to termites. Feng et al. (2022) treated *O. formosanus* with ds*Oftermicins* extracted using the Trizol method, revealing the crucial role of dsRNA in termite defense against infection by SM1, a Gram-negative bacterium [27]. In addition, we also isolated a strain of Gram-negative bacterium Bt and a strain of fungi *Beauveria bassiana* (Bb) from dead *O. formosanus,* which were highly virulent to termites [28]. However, it remains unknown whether ds*Oftermicins* can enhance the virulence of Bt and Bb to termites. Additionally, obtaining high-purity dsRNA via the Trizol method poses challenges and may not be practical for numerous real-world applications. Therefore, we established an inactivated dsRNA-HT115 expression system to produce ample quantities of *O. formosanus* dsRNAs. To enhance its efficacy, we co-associated the expressed ds*Oftermicins* with the Gram-positive bacterium *Bacillus thuringiensis* (Bt) or the fungus *Beauveria bassiana* (Bb). This study offers a theoretical foundation for the application of ds*Oftermicins* in biological control and contributes to the advancement of biological control methods.

## 2. Materials and Methods

### 2.1. Test Insects and Strains

*O. formosanus* were collected from Nanjing, Jiangsu Province, China, and were identified by soldiers, and subsequently reared in a controlled facility with dedicated breeding and rearing areas. Fermented sawdust was supplied under conditions of complete darkness, with a relative humidity maintained at 90 ± 5% and a temperature of 25 ± 1 °C.

For sample preparation, 1 μL of preserved Bt strain was inoculated into LB medium and incubated at 37 °C with agitation at 180 rpm for one week to facilitate the observation of companion cell crystals under the microscope (Olympus Corporation, Beijing, China). The Bb strain was cultured on PDA medium for two weeks at 30 °C. Subsequently, Bb spores were harvested and washed with 0.1% Tween80 to prepare Bb spore suspensions for subsequent use.

### 2.2. Preparation of dsRNA Using Ultrasonic Inactivation of dsRNA-HT115 Strain

According to Feng et al. [27], the *Oftermicin1* (ON080847) plasmid or *Oftermicin2* (ON080848) plasmid was used as a template, and the designed primers (Table S1) were used to synthesize the dsRNA. The ds*Oftermicins* forward primer and reverse primer included the restriction enzyme cutting sites of Not I and Hind III, respectively; the dsRNA for green fuorescent protein [29] (ds*GFP*) forward primer and reverse primer inlcuded the restriction enzyme cutting sites of Sac I and Hind III, respectively. The PCR products were cloned into the RNAi expression vector L4440 (Tsingke Biotechnology Co., Ltd., Nanjing, China). The dsRNA-HT115 strains expressing ds*Oftermicins* or ds*GFP* were stored at −80 °C, Nanjing Forestry University, China.

To prepare the bacterial solution, 1 μL of preserved ds*Oftermicin1*-HT115 was added to 50 mL of LB medium supplemented with 50 μL of ampicillin (100 μg/mL) and 125 μL of tetracycline (12.5 μg/mL). The resulting mixture was then incubated overnight. For the subsequent step, 8 mL of LB medium, supplemented with 8 μL of ampicillin (102.5 μg/mL) and 20 μL of tetracycline (12.8 μg/mL), was transferred to a 10 mL centrifuge tube. Subsequently, 200 μL of the overnight culture medium was added to the tube, followed by incubation at 180 rpm at 37 °C for 2–3 h. Once the OD_600nm_ reached 0.6, 64 μL of IPTG (final concentration of 0.8 mM) was introduced into the culture medium. The medium was subsequently incubated for more than 4 h until the dsRNA expression reached an OD_600nm_ of 1.0.

A volume of 5 mL of the induced ds*Oftermicin1*-HT115 bacterial suspension was centrifuged at 15,984× *g* for 5 min. Subsequently, the supernatant was discarded, and 5 mL of DEPC water was added and thoroughly mixed. Next, the cell suspension was disrupted utilizing an ultrasonic cell crusher operating at 50% power. At 20 min intervals, 50 μL of the bacterial solution was sampled and plated on LB solid medium supplemented with 100 μg/mL ampicillin and 12.5 μg/mL tetracycline. Subsequently, the plates were incubated consistently at 28 °C for 24 h and subsequently observed. The bacterial cultures of ds*Oftermicin2*-HT115 and ds*GFP*-HT115 underwent identical treatment procedures.

### 2.3. Establishment of dsRNA Standard Curves

The dsRNA-HT115 bacterial suspension induced by IPTG was centrifuged at 15,984× *g* at 4 °C for 5 min. Following this, the supernatant was discarded, and TaKaRa RNAiso Plus (TaKaRa Biotechnology Co., Ltd., Nanjing, China) was introduced. After allowing it to stand for 5 min, chloroform was added and vigorously shaken for 15 s (liquid:TaKaRa RNAiso Plus:chloroform = 50:5:1). The sample was then left to stand for an additional 5 min and subsequently centrifuged at 12,787× *g* for 15 min. As a result, the liquid separated into layers, with the top layer removed and mixed with an equal volume of isopropanol. After standing for 10 min, the mixture underwent another round of centrifugation at 12,787× *g* for 10 min at 4 °C. The supernatant was once again discarded, and 80% ethanol was applied to cleanse the sedimentation. The solution was later centrifuged at 12,787× *g* for 5 min, followed by the removal of ethanol. An appropriate quantity of RNase-Free water (Tsingke Biotechnology Co., Ltd., Nanjing, China) was employed to dissolve the precipitate. Subsequently, the concentration of dsRNA was determined by assessing the UV absorbance at 260 nm. The concentrations of dsRNA were set at 2000, 200, 20, and 2 ng/μL. To establish the standard curves for ds*Oftermicin1*, ds*Oftermicin2*, and ds*GFP*, the logarithm of the dsRNA concentration and the CT values obtained through real-time fluorescence quantitative PCR (qRT-PCR) were utilized. Specific primers detailed in (Table S1) were employed for expression [27]. Additionally, the concentration of double-stranded RNA (dsRNA) in the inactivated bacterial fluid was quantified using an absolute quantitative method by correlating the CT value with the standard curve. This concentration was subsequently utilized in the ensuing RNAi experiments.

### 2.4. Interference Efficiency of dsRNA

In this study, we adhered to the procedure outlined by Feng et al. [27]. Dry filter paper was laid out in sterilized Petri dishes measuring 7 cm in diameter, with 20 healthy *O. formosanus* workers being placed in each dish. The filter paper was then treated with a mixture of approximately 500 μL of nile blue (1% *w*/*v*) and ds*Oftermicin1* (0.5 μg/μL). To serve as the positive control, another group was treated with a mixture of 500 μL of nile blue (1% *w*/*v*) and dsGFP (0.5 μg/μL), while a group treated with a mixture of 500 μL of nile blue (1% *w*/*v*) and DEPC water acted as the negative control. Each experiment was independently repeated three times. The termites were situated in a dark environment at room temperature, and those whose bodies turned blue within 12 h were collected for subsequent RNA extraction. The expression levels of *Oftermicin1* and *Oftermicin2* were assessed using qRT-PCR, with glyceraldehyde-3-phosphate dehydrogenase (*GAPDH*) and ribosomal protein S18 (*RPS18*) serving as reference genes. Specific primers detailed in Table S1 [27] were utilized for expression. The qRT-PCR data were analyzed employing the 2^−ΔΔCT^ method [30].

### 2.5. Virulence of dsOftermicin1 and dsOftermicin2 in Combination with Two Pathogenic Bacteria to O. formosanus

In the experimental setup, twenty *O. formosanus* workers were placed in each dish, with dry filter paper laid out in a 7 cm Petri dish. The filter papers were then treated with 400 μL of nile blue (1% *w*/*v*) and ds*Oftermicin1* (0.5 μg/μL) (treatment group), 400 μL of nile blue (1% *w*/*v*) and ds*GFP* (0.5 μg/μL) (positive control), or 400 μL of nile blue (1% *w*/*v*) with DEPC water (negative control). The termites were left to feed for a period of 12 h. After this feeding period, workers from the negative control, positive control, and treatment groups were divided into two groups (uninfected and bacterial infection groups), respectively. Two microliter of culture medium was placed on the worker’s pronotum (control group), and two microliters of pathogenic bacterial suspensions of Bt (1 × 10^8^ cell/mL) and Bb (1 × 10^8^ spore/mL) were dropped on placed on each worker’s pronotum (treatment group). Each experiment was repeated independently three times. The mortality of the termites was monitored and recorded at 3 h intervals. Experimental procedures related to ds*Oftermicin2* were conducted following the same protocols as described above.

### 2.6. Statistical Method

The InStat software version 3.05 was employed to perform analysis of variance (ANOVA). For comparisons between two samples, a two-tailed unpaired Student’s *t*-test was used to assess significant differences. When conducting multiple sample comparisons, the statistical significance was evaluated using one-way ANOVA with Tukey’s multiple comparisons post hoc test. In this study, a *p*-value of less than 0.05 was considered statistically significant, indicating that the observed differences were unlikely to have occurred by random chance.

## 3. Results

### 3.1. Construction of Inactivated dsRNA-HT115 System for dsRNA Production

#### 3.1.1. Acquisition of Inactivated ds*Oftermicin1*-HT115 and ds*Oftermicin2*-HT115

**Figure 1:**
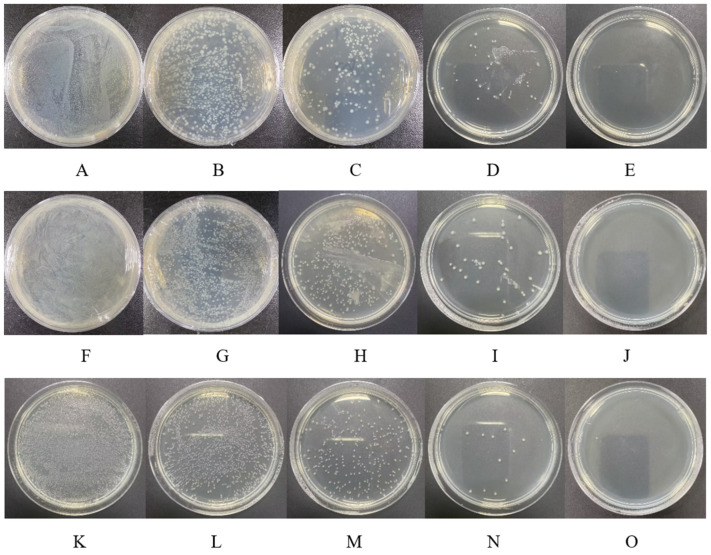
Plate coating results of bacterial solution at different times of ultrasonic crushing. (**A**–**E**): ds*Oftermicin1*-HT115 was crushed for 0 h, 0.5 h, 1 h, 1.5 h, and 2 h, respectively. (**F**–**J**): ds*Oftermicin2*-HT115 was crushed for 0 h, 0.5 h, 1 h, 1.5 h, and 2 h, respectively. (**K**–**O**): ds*GFP*-HT115 was crushed for 0 h, 0.5 h, 1 h, 1.5 h, and 2 h, respectively.

The induced bacterial solution was sonicated using an ultrasonic cell crusher, and samples were collected every 30 min and coated on solid LB medium. It was observed that the number of bacterial colonies decreased with increasing sonication time, and no more bacterial colonies appeared on the medium after 2 h of sonication (Figure 1). The bacterial solutions at this time were used for subsequent experiments.

#### 3.1.2. Standard Curve of dsRNA in *O. formosanus*

**Figure 2:**
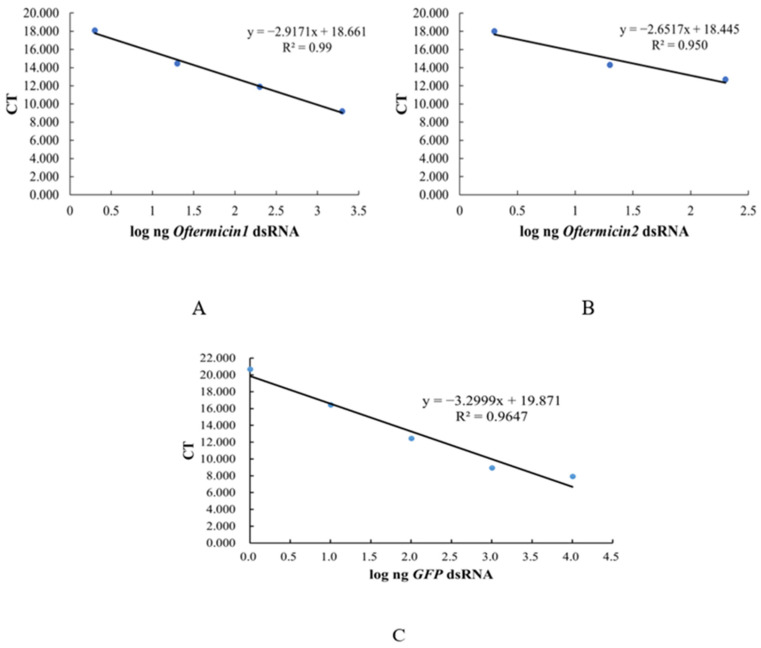
Standard curve for dsRNA from inactivated dsRNA-HT115 bacterial solutions. (**A**) ds*Oftermicin1*; (**B**) ds*Oftermicin2*; (**C**) ds*GFP.*

The CT values obtained from qRT-PCR were plotted against the logarithm of the four gradient concentrations of dsRNA to establish the standard curve. The standard curve for ds*Oftermicin1* was calculated as y = −2.917x + 18.661 (R^2^ = 0.993) (Figure 2A), and for ds*Oftermicin2*, it was y = −2.6517x + 18.445 (R^2^ = 0.9506) (Figure 2B). The standard curve for ds*GFP* was determined to be y = −3.2999x + 19.871 (R^2^ = 0.9647) (Figure 2C).

#### 3.1.3. Interference Efficiency of dsRNA

**Figure 3:**
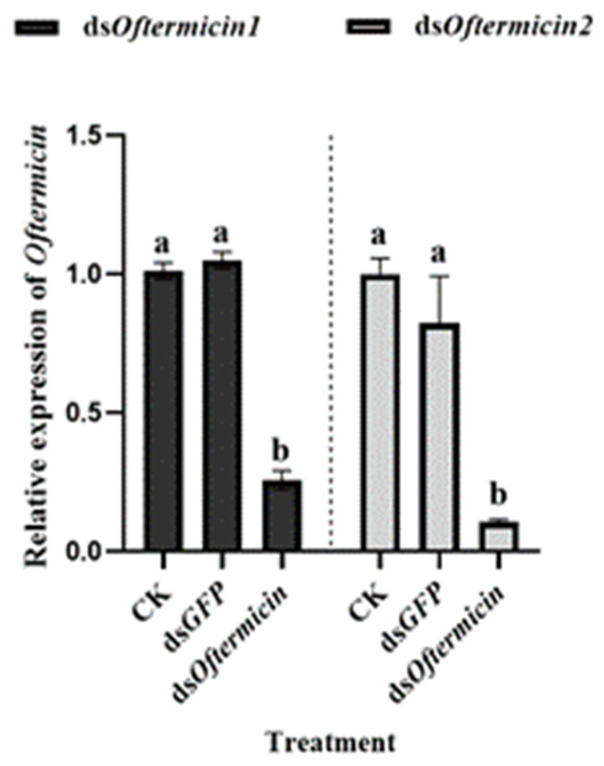
Effect of ds*Oftermicin1* and ds*Oftermicin2* on the relative expression of *Oftermicin1* and *Oftermicin2* in *O. formosanus*. Negative control (CK) was ddH_2_O treatment and positive control (ds*GFP)* was ds*GFP* treatment, and ds*Oftermicin1* and *Oftermicin2* was dsRNA treatment. The values were given as mean ± SD of three replicate samples. Significant difference in mortality rate was indicated by the different letters on the error bars (*p* < 0.05).

The workers of *O. formosanus* were treated with ds*Oftermicin1* and ds*Oftermicin2* for 12 h, and samples were collected. qRT-PCR was used to detect the expression of the *Oftermicin1* and *Oftermicin2* genes. It was observed that the gene expression of both *Oftermicin1* and *Oftermicin2* was suppressed at 12 h, with their transcriptional levels decreasing by 74.31% and 89.46%, respectively (Figure 3).

### 3.2. Virulence of dsOftermicin1 and dsOftermicin2 in Combination with Two Pathogenic Bacteria to O. formosanus

#### 3.2.1. Virulence of dsRNA Coupled with Bt to *O. formosanus*

**Figure 4:**
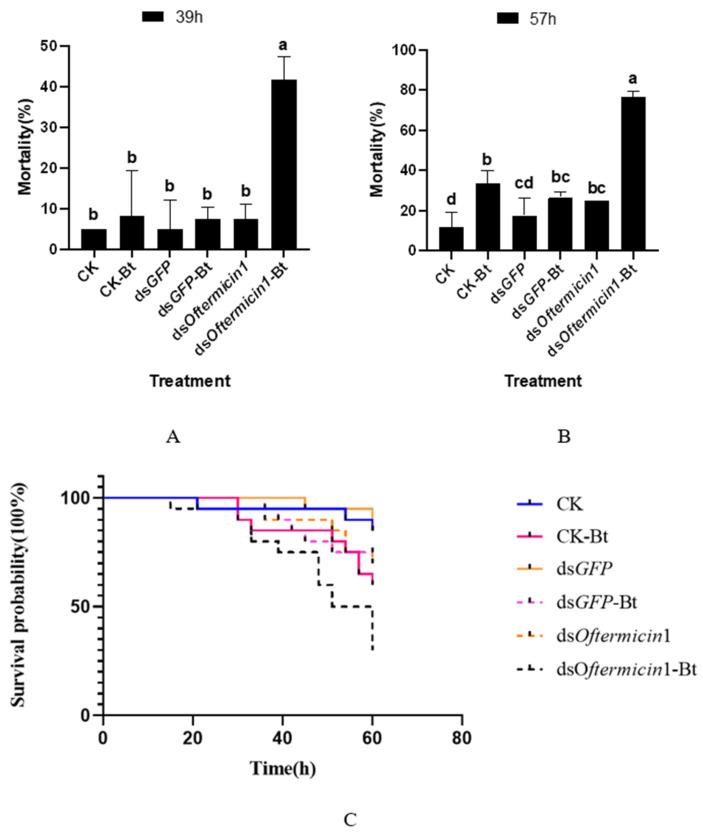
Virulence of ds*Oftermicin1* in combination with Bt to *O. formosanus*. (**A**) Mortality in each treatment group at 39 h. (**B**) Mortality in each treatment group at 57 h. (**C**) Survival rates for each treatment group. CK: ddH_2_O treatment; ds*GFP:* ds*GFP* treatment; ds*Oftermicin1*: dsRNA treatment; CK-Bt: ddH_2_O and Bt treatment; ds*GFP-*Bt: ds*GFP* and Bt treatment; ds*Oftermicin1*-Bt: dsRNA and Bt treatment. The values were given as mean ± SD of three replicate samples. Significant difference in mortality rate was indicated by the different letters on the error bars (*p* < 0.05).

After silencing the *Oftermicin1* gene and treating *O. formosanus* with Bt, it was observed that at 39 h, the mortality rate of the ds*Oftermicin1*-Bt group (41.67%) was significantly higher than that of the other treatment groups (Figure 4A). Furthermore, at 57 h, the mortality rate of the ds*Oftermicin1*-Bt group reached its peak at 76.67%, which was significantly higher than that of the CK-Bt group (33.75%) and the ds*GFP*-Bt group (26.67%) (Figure 4B). The survival curve results indicated that the survival rate of the ds*Oftermicin1*-Bt group was significantly lower than that of the other Bt treatment groups (CK-Bt and ds*GFP*-Bt) (Figure 4C).

**Figure 5:**
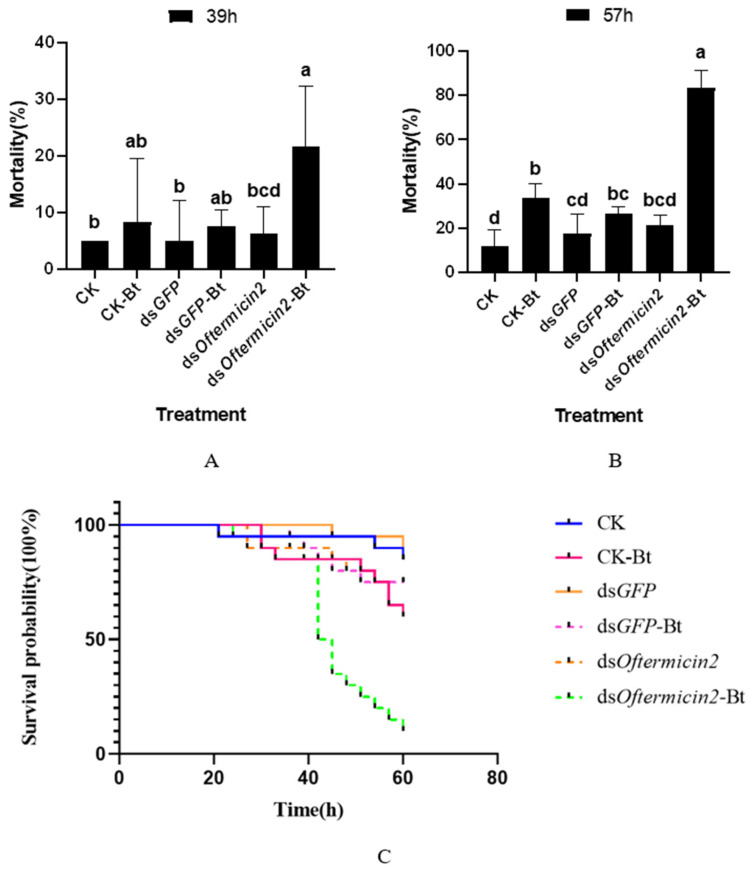
Virulence of ds*Oftermicin2* in combination with Bt to *O. formosanus*. (**A**) Mortality in each treatment group at 39 h. (**B**) Mortality in each treatment group at 57 h. (**C**) Survival rates for each treatment group. CK: ddH_2_O treatment; ds*GFP:* ds*GFP* treatment; ds*Oftermicin2*: dsRNA treatment; CK-Bt: ddH_2_O and Bt treatment; ds*GFP-*Bt: ds*GFP* and Bt treatment; ds*Oftermicin2*-Bt: dsRNA and Bt treatment. The values were given as mean ± SD of three replicate samples. Significant difference in mortality rate was indicated by the different letters on the error bars (*p* < 0.05).

After silencing the *Oftermicin2* gene and treating *O. formosanus* with Bt, it was observed that at 39 h, the mortality rate of the ds*Oftermicin2*-Bt group (21.67%) was significantly higher than that of the other treatment groups (Figure 5A). Furthermore, at 57 h, the mortality rate of the ds*Oftermicin2*-Bt group reached its peak at 83.33%, which was significantly higher than that of the CK-Bt group and the ds*GFP*-Bt group (Figure 5B). The survival curve results indicated that the survival rate of the ds*Oftermicin2*-Bt group was significantly lower than that of the other Bt treatment groups (CK-Bt and ds*GFP*-Bt) (Figure 5C).

#### 3.2.2. Virulence of dsRNA Coupled with Bb to *O. formosanus*

**Figure 6:**
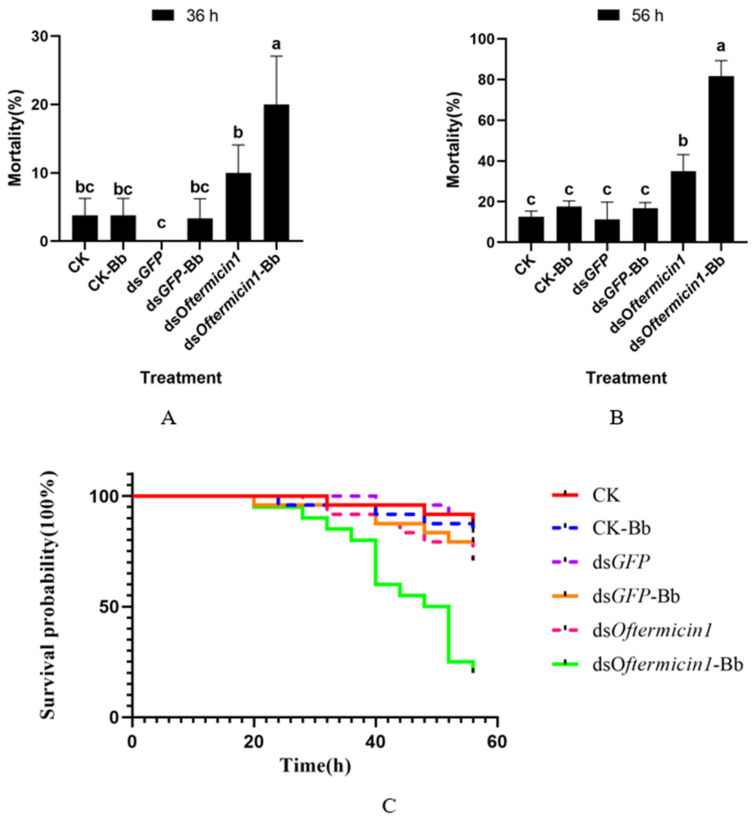
Virulence of ds*Oftermicin1* in combination with Bb to *O. formosanus*. (**A**) Mortality in each treatment group at 36 h. (**B**) Mortality in each treatment group at 56 h. (**C**) Survival rates for each treatment group. CK: ddH_2_O treatment; ds*GFP:* ds*GFP* treatment; ds*Oftermicin1*: dsRNA treatment; CK-Bb: ddH_2_O and Bb treatment; ds*GFP-*Bb: ds*GFP* and Bb treatment; ds*Oftermicin1*-Bb: dsRNA and Bb treatment. The values were given as mean ± SD of three replicate samples. Significant difference in mortality rate was indicated by the different letters on the error bars (*p* < 0.05).

After silencing the *Oftermicin1* gene and treating *O. formosanus* with Bb, the results showed that at 36 h, the mortality rate of the ds*Oftermicin1*-Bb group (20%) was significantly higher than that of the other treatment groups (Figure 6A). Furthermore, at 56 h, the mortality rate of the ds*Oftermicin1*-Bb group reached its peak at 81.67%, which was significantly higher than that of the CK-Bb group (17.5%) and the ds*GFP*-Bb group (16.66%) (Figure 6B). The survival curve results indicated that the survival rate of the ds*Oftermicin1*-Bb group was significantly lower than that of the other Bb treatment groups (CK-Bb and ds*GFP*-Bb) (Figure 6C).

**Figure 7:**
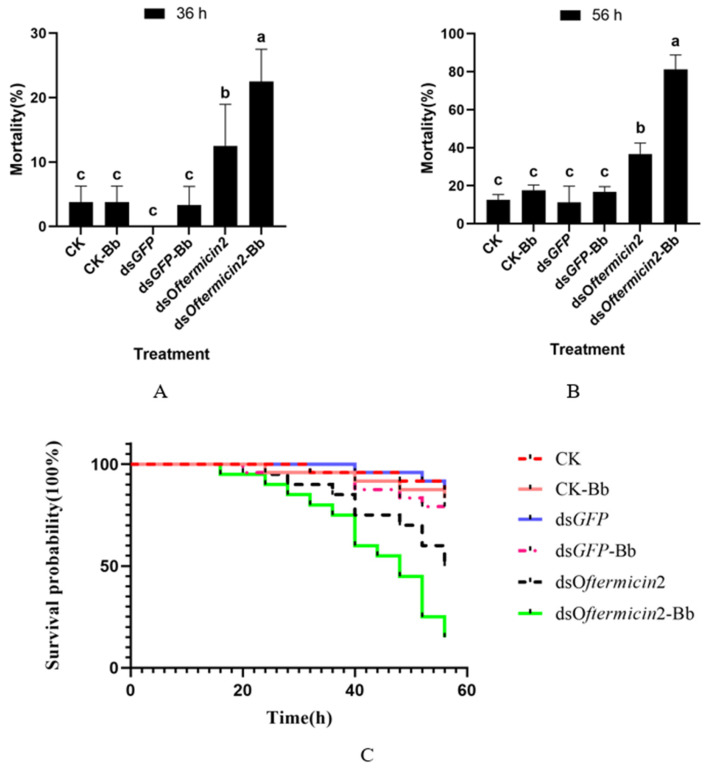
Virulence of ds*Oftermicin2* in combination with Bb to *O. formosanus*. (**A**) Mortality in each treatment group at 36 h. (**B**) Mortality in each treatment group at 56 h. (**C**) Survival rates for each treatment group. CK: ddH_2_O treatment; ds*GFP:* ds*GFP* treatment; ds*Oftermicin2*: dsRNA treatment; CK-Bb: ddH_2_O and Bb treatment; ds*GFP*-Bb: ds*GFP* and Bb treatment; ds*Oftermicin2*-Bb: dsRNA and Bb treatment. The values were given as mean ± SD of three replicate samples. Significant difference in mortality rate was indicated by the different letters on the error bars (*p* < 0.05).

After silencing the *Oftermicin2* gene and treating *O. formosanus* with Bb, it was observed that at 36 h, the mortality rate of the ds*Oftermicin2*-Bb group (22.5%) was significantly higher than that of the other treatment groups (Figure 7A). Additionally, at 56 h, the mortality rate of the ds*Oftermicin2*-Bb group reached its peak at 81.25%, which was significantly higher than that of the CK-Bb group and the ds*GFP*-Bb group (Figure 7B). The survival curve results indicated that the survival rate of the ds*Oftermicin2*-Bb group was significantly lower than that of the other Bb treatment groups (CK-Bb and ds*GFP*-Bb) (Figure 7C).

## 4. Discussion

Current biological control primarily relies on the utilization of natural predators and microbial agents [5]. Numerous researchers have conducted experiments involving pathogenic bacteria in termites. Following assessments of various entomopathogenic fungi and bacteria, Toumanoff and Rombaut (1965) identified Ma and Bb as the two most virulent entomopathogenic microorganisms against *Reticulitermes flavipes* [31]. Additionally, Wang and Powell (2003) compared the virulence of six strains of Bb and two strains of Ma to *R. flavipes*, with their findings indicating that the virulence of Bb was significantly lower than that of Ma [32]. Tamashiro (1976) proposed investigating the effects of various pathogens on *Coptotermes formosanus*, including nematodes (*genus Steinernema*) and fungi (Ma and Bb). However, their preliminary data indicated that despite promising in-laboratory bioassays, field trials did not yield positive results [33]. In 1996, Suzuki conducted a study to test the effectiveness of three pathogenic fungi (Ma, Bb, and *Paecilomyces fumosoroseus*) against *C. formosanus*. Indoor tests confirmed the virulence of all three fungi to *C. formosanus*, with Ma being the most effective. However, the field tests did not yield satisfactory results [34]. Additionally, tests were conducted on worker termites of *Heterotermes indicola* and *Bifiditermes beesoni* using Bt and SM. The results showed that the mortality rate of the termites could reach 100% within 6–13 days and 7–13 days respectively. However, none of the field experiments yielded satisfactory results [35]. Indeed, social insects are seldom affected by pathogens in their environment owing to their robust innate immune system [36]. This enables them to eliminate pathogens through cellular immunity, activate Toll, IMD, and JAK-STAT pathways, and subsequently induce relevant immune responses to pathogen invasion [37]. Additionally, certain social immune behaviors of termites can also influence pathogen infestation [11]. Furthermore, genomic studies of social insects have revealed that termites and cockroaches possess a complete immune gene pool [38,39,40], equipping them to defend against external threats. This renders biological control of termites less effective and more challenging in practice. Fortunately, in recent years, the emergence of RNAi technology has addressed this dilemma. RNAi links phenotypes to gene functions, enabling researchers to analyze and regulate target gene functions by suppressing their expression [41]. The integration of RNAi technology with pathogenic bacteria holds significant potential for the biological control of termites. This approach also presents novel strategies for termite control.

Termite innate immunity involves the synthesis and release of antimicrobial peptides (AMPs) in response to pathogens. These peptides contain bioactive components that inhibit or directly kill pathogens [42,43]. AMP identified in termites include termicin and spinigerin. Termicin affects the growth of various fungi by inhibiting spore germination or mycelial perforation, and it also inhibits the activity of certain Gram-positive bacteria. The helical structure spanning from Lys4 to Thr23 of spinigerin is also antibacterial. The electrostatic attraction between lysine and arginine and the negatively charged polar head of the phosphate can disrupt the cell membrane, leading to bacterial death. Therefore, utilizing termicin and spinigerin as target genes for RNA interference (RNAi) will undoubtedly elucidate their roles in termite humoral immunity. Bulmer et al. (2009) investigated *GNBP-2* derived from *Nasutitermes cornigera* and discovered that *GNBP* exhibits β-1,3-glucanase activity, serving as a recognition receptor [44]. *GNBPs* can utilize their β-1,3-glucanase activity to disrupt the fungal cell wall and spore wall, enabling smaller effector molecules (such as AMPs) to penetrate the cell, ultimately leading to the complete destruction of the fungal cell [45]. Building upon this research, Hamilton and Bulmer (2012) isolated double-stranded RNAs (dsRNAs) targeting *termicin* and *GNBP2* from *R. flavipes.* Their experiments revealed a notable reduction in the antimicrobial activity of termite epidermis and an elevation in termite mortality upon exposure to Ma [45]. In our laboratory, Feng et al. [27]. successfully enhanced the virulence to *O. formosanus* by utilizing pure ds*Oftermicins* acquired through the Trizol method in conjunction with SM1, a Gram-negative bacterium. However, as SM1 is Gram-negative, the study did not investigate whether ds*Oftermicins* could boost the virulence of Gram-positive bacteria and fungi to *O. formosanus.* To address this gap, we conducted a study combining ds*Oftermicins* with both a gram-positive bacterium (Bt) and a fungus (Bb). The findings revealed that ds*Oftermicins* not only amplified the virulence of Bt but also enhanced the virulence of Bb. Furthermore, we observed that the dsRNA extraction process described by Feng et al. [27]. was complex and associated with high economic and time expenditures, rendering it impractical for widespread application. Moreover, urgent attention is required to address the issue of dsRNA degradation. Building upon these observations, we established a novel system for obtaining dsRNA from transgenic strains inactivated via ultrasonic fragmentation, a first in *O. formosanus*. The in vitro synthesis of ds*Oftermicins* utilizing the bacterium HT115 (DE3) not only yielded a substantial quantity of dsRNA rapidly, but also, owing to the disrupted bacterial cell membranes, safeguarded the dsRNA from degradation. Furthermore, since transgenic strains cannot be directly utilized, we implemented the ultrasonic crushing method within our system to pulverize the dsRNA-expressing strains prior to use, thereby mitigating this limitation. This advancement holds significant implications for the practical deployment of biocontrol measures. Our study demonstrated that ds*Oftermicin1* and ds*Oftermicin2* significantly enhanced the virulence of Bt and Bb to *O. formosanus*. These findings provide a theoretical basis for the research and development of termite RNA biopesticides, and they contribute to the progress and implementation of biological control strategies targeting termites.

## 5. Conclusions

In conclusion, the study findings suggest that administering ds*Oftermicin1* and ds*Oftermicin2* derived from inactivated engineered bacteria to termites effectively suppressed the expression of *Oftermicin1* and *Oftermicin2* genes in *O. formosanus*. Additionally, the study demonstrated that ds*Oftermicin1* and ds*Oftermicin2* could augment the virulence of Bt and Bb to *O. formosanus*. Therefore, *Oftermicin1* and *Oftermicin2* play a crucial role in *O. formosanus* resistance against pathogens and represent essential target genes for RNAi-based termite control.

## Data Availability

The original contributions presented in the study are included in the article/Supplementary Materials, further inquiries can be directed to the corresponding author.

## Supplementary Materials

The following supporting information can be downloaded at: https://www.mdpi.com/article/10.3390/insects15050360/s1, Table S1: Primers used in this study [27].
