# Supplementary material for: The Function of Termicin from Odontotermes formosanus (Shiraki) in the Defense against Bacillus thuringiensis (Bt) and Beauveria bassiana (Bb) Infection
Source: Insects. 2024 May 16;15(5):360. doi: 10.3390/insects15050360 (PMC11122213; doi:10.3390/insects15050360)
Supplement: Supplementary file 1 [file insects-15-00360-s001.zip › insects-2995403-supplementary.pdf]

Table S1. Primers used in this study [27].

Gene Name	Orientation	(5′ → 3′) Primer Sequence	Purpose
Oftermicin1	Forward	*ATTT***GCGGCCGC**AGGACTGTCGCTAT	RNAi
	Reverse	*CCC***AAGCTT**GCCCCCAGTAACATACA	
	Forward	CTAGTCTTCCTGGTAGTGGTTTG	Internal references and standard curves for qRT-PCR
	Reverse	CAAGTGGGTCCATCACAGAA	
Oftermicin2	Forward	*ATTT***GCGGCCGC**AAGACCGTTACTAGCCTACTTGT	RNAi
	Reverse	*CCC***AAGCTT**GCCGTTAGGATTATACACACAT	
	Forward	CCGTTACTAGCCTACTTGTCTTT	Internal references and standard curves for qRT-PCR
	Reverse	GTCCATCACAGAACGCTCTTA	
dsGFP	Forward	*ATC***GGAGCT**CAGTTGAACGGATCCATCTTCA	RNAi and standard curves for qRT-PCR
	Reverse	*CCC***AAGCTT**AGAACTTTTCACTGGA	
RPS18	Forward	ATGGCAAACCCCCGTCAGTA	Internal references for qRT-PCR
	Reverse	CATACCACGATGCGCACGAA	
GAPDH	Forward	TCGTATTGGCCGTCTTGTGC	Internal references for qRT-PCR
	Reverse	AGCGACCATGGGTGGAATCAT	

Note: The primer underline is the restriction site, and 3–4 letters in front of the underline are protective bases.
